# Exclusion of SARS-COV-2 From Two Maine Overnight Camps July-August 2020

**DOI:** 10.1017/dmp.2021.86

**Published:** 2021-03-25

**Authors:** Rachel H. Kowalsky, Susan Fine, Matthew A. Eisenberg

**Affiliations:** 1 Departments of Emergency Medicine and Pediatrics, New York Presbyterian Hospital-Weill Cornell Medicine, New York, New York, USA; 2 Department of Internal Medicine and Pediatrics, University of Massachusetts, Worcester, Massachusetts, USA; 3 Division of Emergency Medicine, Boston Children’s Hospital, Boston, Massachusetts, USA

**Keywords:** summer camp, nonpharmaceutical intervention, COVID-19

## Abstract

**Objectives:**

Summer camp can positively affect self-esteem and social skills. Most United States summer camps did not open during 2020 because of concerns about severe acute respiratory syndrome coronavirus 2 (SARS-COV-2). Our objective is to describe exclusion strategies successfully used by 2 summer camps in Maine.

**Methods:**

Before camp arrival, all attendees were asked to quarantine at home for 14 d and perform a daily symptom checklist. Salivary specimens were submitted by mail for SARS-COV-2 PCR testing 4 d before arrival, and again 4 d after arrival. At camp, multiple layers of nonpharmaceutical interventions (NPIs) were used.

**Results:**

A total of 717 (96.7%) prospective attendees underwent remotely supervised saliva collection; 4 were positive and did not come to camp. Among the 20 who did not submit a sample, 3 did not come to camp; the other 17 underwent screening and a rapid antigen test for SARS-COV-2 immediately upon arrival and before reporting to communal living spaces; all were negative. All campers and staff were re-tested by salivary polymerase chain reaction 4 d after arrival, and all were negative.

**Conclusions:**

We demonstrate that it is possible to safely operate overnight camps during a pandemic, thus supporting the continued physical and socioemotional growth of children, using multiple layers of NPIs.

Summer camp can positively affect self-esteem, independence, and social skills.^1^ The majority of United States overnight summer camps did not open during the summer of 2020 because of concerns about potential severe acute respiratory syndrome coronavirus 2 (SARS-COV-2) outbreaks.^2,3^ Those that opened had varied experiences excluding and containing SARS-COV-2,^4,5^ with successful camps reporting rigorous pre-entry quarantine and testing protocols, and multiple layers of nonpharmaceutical interventions (NPIs).^5^ We describe 2 affiliated Maine overnight summer camps that successfully excluded SARS-COV-2.

## Methods

This study was approved with a waiver of informed consent by the Boston Children’s Hospital’s Institutional Review Board. Maine entered Stage 3 of their reopening plan on July 1, 2020, allowing residential overnight camps to open with precautions in place.^6^ Both camps, an affiliated girls’ camp and boys’ camp, opened from July 10 until August 14, 2020. They adhered to guidance from the Centers for Disease Control^7^ and the American Academy of Pediatrics,^8^ summarized in a field guide by the American Camp Association,^9^ and followed rules established by the Maine Department of Public Health. NPI’s were executed in 3 phases: (1) precamp quarantine and testing, and cohorting by cabin (“family”); (2) cohorting by age group; and (3) extended cohorting. See Table 1 for NPI’s from time relative to camp start date.

**Table 1.** Non-Pharmaceutical Interventions by Time Relative to Camp Start

Time relative to camp start	NPI
1 m. prior	- Staff completed daily clinical screen and isolated at home x 14 days
15 d. prior	- Staff arrived at camp - Campers isolated at home x 14 days
11 d. prior	- All staff tested for SARS-CoV-2
7 d. prior	- Campers completed daily clinical screen
4 d. prior	- All campers tested for SARS-CoV-2
Day 0	- Travel protocols
Day 4	- Second SARS-CoV-2 test
Day 7-35	- Cohorting - Activity modification - Equipment sanitization - Frequent hand hygiene - Daily temperature checks - Packaged foods, sanitized on delivery - No out of camp trips or visitors - Rapid testing and isolation of any ill camper - Weekly testing of day staff

### Phase 1

There were 2 camp entry dates: 1 for staff, and one 14 d later for campers. The traditional second camp session was eliminated to avoid multiple entry dates. Two weeks before their respective entry date, attendees were asked to quarantine at home and complete a daily symptom checklist, which was signed and submitted to the camp infirmary. Four days before their entry date, attendees underwent salivary SARS-COV-2 polymerase chain reaction (PCR) testing. Specimens were obtained by means of remotely supervised collection and submitted by mail (Vault Health, New York, NY).

Travel to camp was carefully planned and undertaken. Those who traveled by airplane were directed to wear masks at all times. For bus travel, all buses were limited to 50% capacity. Families remained in their cars except for 1 family member who accompanied each camper to the bus. Campers underwent temperature checks before boarding, and wore masks while onboard. The first 2 rows were left vacant to allow distance from the driver. No stops were made en route to camp. Arrivals by car were assigned staggered arrival times. Temperature checks and a symptom checklist were conducted before campers joined their groups.

Upon arrival, campers were divided into “families” by cabin, and traveled to all activities by family. Families were identified by colored bracelets. Masks were worn whenever there was potential for mixing with another family indoors, for example in the dining hall.

All activities were modified to allow for equipment sanitization and frequent hand hygiene. Daily temperature checks were conducted using a noncontact infrared thermometer. When possible, food was prepackaged and sanitized on delivery. No out of camp trips took place, and no visitors were allowed to campus. All staff lived on the premises except for a small group of day workers, who underwent weekly rapid antigen testing. Campers with any signs of viral illness (fever, cough, sore throat, emesis, or diarrhea) underwent rapid antigen testing and were isolated until their symptoms resolved. Isolation took place either in the infirmary, or at an adjoining camp that had not opened for the summer and had contracted to provide living space for this purpose.

Four days after arriving at camp, all attendees repeated the salivary PCR test. When results were available and negative, Phase 2 began.

### Phase 2

Campers traveled by age cohort. They wore masks whenever there was potential for mixing with another cohort indoors. All activity modifications from phase 1 continued through phases 2 and 3.

### Phase 3

When 14 d had elapsed with no identified SARS-COV-2 cases at camp, attendees were permitted to unmask most of the time. Masks were still required indoors when age cohorts mixed.

Counselors were considered part of the cabin (family) where they lived. They wore masks whenever there was potential for mixing with persons outside their family or cohort. Counselors who did not live with campers did not enter any camper cabins in phase 1, and were socially distant and masked during activities in phases 1 and 2.

Three physicians (M.E., R.K., S.F.) provided care at both camps sequentially.

## Results

Of the 738 campers, staff, and family of staff planning to attend camp, 435 (58.9%) were male and 303 (41.1%) were female. Most (464/738; 62.9%) were campers. The most common regions of origin were New England (35.9%), the Middle Atlantic (24.5%), and the South (24.3%). See Table 2 for subject characteristics.

**Table 2.** Characteristics of Attendees, Two Maine Overnight Camps, July-August 2020

Characteristic	No. (%)
Total	738
**Sex**	
M	435 (58.9)
F	303 (41.1)
**Role**	
Camper	464 (62.9)
Staff Member - residential	262 (35.5)
Staff Member – day staff	9 (1.2)
Child of staff member	3 (0.4)
**Age Group, yrs**	
<7	4 (0.5)
7-8	34 (4.6)
9-10	125 (16.9)
11-12	140 (19)
13-14	110 (14.9)
15-18	88 (11.9)
19-21	105 (14.2)
22-29	83 (11.2)
30-49	36 (4.9)
50-70	13 (1.8)
**Home Region**^1^	
Middle Atlantic	181 (24.5)
South	179 (24.3)
New England	265 (35.9)
Midwest	35 (4.7)
West Coast	72 (9.8)
International	6 (0.8)
**Mode of Travel**	
Bus	164 (22.2)
Car	378 (51.2)
Plane – commercial	123 (16.7)
Plane - charter	65 (8.8)
Did not come	8 (1.1)

1 Regions defined by “Census Regions and Divisions of the United States” https://www2.census.gov/geo/pdfs/maps-data/maps/reference/us_regdiv.pdf

Before camp arrival, 717 (96.7%) campers underwent remotely supervised saliva collection; 4 were positive and did not come to camp. Among the 20 who did not submit a sample, 3 did not come to camp; the other 17 underwent screening and a rapid antigen test for SARS-COV-2 immediately upon arrival and before reporting to communal living spaces; all were negative.

No campers or staff were excluded based on the precamp or arrival day symptom screens. One child had a fever during temperature screening at the bus to camp. His arrival was delayed by 1 d, until he had a negative PCR test and had been afebrile and asymptomatic for the intervening 24 h.

All campers and staff were re-tested by salivary PCR 4 d after arrival, and all were negative. During the 5-wk camp session, 65 people had at least one additional test (PCR or antigen) while at camp, either for weekly testing of day staff, or for illness. PCRs were obtained to confirm negative rapid tests at the discretion of the camp physicians. All tests were negative.

### Limitations

While it was successful, the protocol described here has several limitations that may impact its reproducibility.

First, both sleepaway camp and serial SARS-COV-2 testing are costly, limiting access. Second, the majority of campers came from New England and the Middle Atlantic, regions of the country with a low prevalence of SARS-COV-2 during summer 2020,^4^ which likely played a role in excluding the virus.

The use of antigen testing as a screen is known to have limited accuracy in asymptomatic people. This method was used for weekly testing of day workers because of its rapidity and ease, given the isolated rural setting. The low community prevalence in Maine during summer 2020 (less than 3%)^10^ mitigated the likelihood of false negative results.

NPI’s are most effective when compliance is high. The 2-wk isolation period was likely key in the low number of pretravel positive tests; this could be challenging in groups whose parents cannot work from home, or as pandemic fatigue affects behavior. Similarly, compliance with NPI’s instituted on-site at camp may differ across specific groups and contexts.

Finally, without a postcamp PCR, complete confidence in having excluded SARS-COV-2 cannot be assured, because of the potential for asymptomatic spread. However, there were no reports of infection from campers or staff after returning home.

## Discussion

We have described the experience of 2 Maine overnight summer camps that successfully excluded SARs-COV-2 during July and August 2020. The camps’ ability to insulate themselves from the virus is likely attributable to close adherence to established guidelines, scrupulous testing, and instituting multiple layers of NPI’s.

## Conclusions

We demonstrate that it is possible to safely operate overnight camps during a pandemic, thus supporting the continued physical and social-emotional growth of children, by using multiple layers of NPIs. These findings may help inform future practice for sleepaway camps and other communal living settings such as boarding schools.
